# Metabolic syndrome: new therapeutic approaches

**DOI:** 10.1186/1824-7288-40-S1-A48

**Published:** 2014-08-11

**Authors:** Claudia Della Corte, Valerio Nobili

**Affiliations:** 1 Hepatometabolic Department, “Bambino Gesù” Children’s Hospital, S. Onofrio Square, 4, 00165, Rome, Italy

## 

In the last three decades in the United States the prevalence of overweight/obesity in pediatric population has more than tripled, causing the onset also in pediatric age of diseases previously considered exclusively of adults, such as metabolic syndrome (MetS) [1]. MetS represents a cluster of cardiometabolic abnormalities, including visceral obesity, dyslipidemia, hypertension and diabetes mellitus type 2 (T2DM) (Table 1). The prevalence of pediatric MetS ranged from 2% to 9% in the general population and from 12% to 44% in obese children, depending of definition used [1]. Several evidences suggest that the metabolic derangements observed in children may have a worrisome repercussion early on their health in adulthood [2,3].

**Table 1:** Diagnostic criteria for metabolic syndrome in children and adolescents

	IDF criteria		
**Age (years)**	**6-9**	**10-15**	**>15 (adult criteria)**

Waist circumference	≥90^th^ percentile for age (MS as entity is not diagnosed)	≥90^th^ percentile or adult cut-off if lower	≥94 cm for males, ≥80 cm for females

Blood pressure		Systolic ≥130 or diastolic ≥85 mmHg	Systolic ≥130 or diastolic ≥85 mmHg or treatment of previously diagnosed hypertension

Triglycerides		≥1.7mmol/L (≥150 mg/dL)	≥1.7mmol/l (≥150 mg/dL)or specific treatment for high triglycerides

HDL-C		< 1.03 mmol/L (<40 mg/dL)	< 1.03 mmol/L (<40 mg/dL) in male and 1.29 mmol/L (<50 mg/dL) in females or specific treatment for low HDL-C

Fasting glucose		5.6 mmol/l (100 mg/dL)	5.6 mmol/l (100 mg/dL) or known T2DM

IDF: International Diabetes Federation

HDL Cholesterol: High-density lipoprotein cholesterol

Lifestyle modification, represented by the association among regular physical exercise and a balanced diet appropriate for age, is the most important therapeutic approach in children and adolescent with obesity and risk factors for MetS [4]. Behavioral intervention is mandatory but, in many cases, it is difficult to achieve or not sufficient and most pediatric patients require pharmacologic therapy early in their disease course. At the present time, the vast majority of drugs needed to treat insulin-resistance, hypercholesterolemia, hypertension are off-label in pediatric setting, although several studies demonstrated that pharmacological treatment for pediatric obesity and its related comorbidities are necessary [5].

Regarding dyslipidemia, the use of oral statins is reserved for children older than 10 years of age that, while on diet, continue to have dyslipidemia associated to family history for early cardiovascular disease (CVD) or additional risk factors.

Metformin is the only drug approved for treatment of impaired fasting glucose (IFG) or impaired glucose tolerance (IGT) in children. Several studies demonstrated its efficacy in ameliorating gluco-insulinemic profile; moreover, it has been reported a moderately effect on body weight in obese children.

Anti-hypertensive drugs, such as angiotensin-converting-enzyme inhibitors (ACEIs), angiotensin-receptor blockers (ARBs), calcium channel blockers (CCBs), and beta-blocking agents are used in pediatric patients and the choice of drug class is made on the basis of clinical characteristics of the single patient.

The core of treatment of pediatric MetS is abdominal obesity. Currently no pharmacological approach to obesity is accepted for pediatric patients. Bariatric surgery has been considered a successful treatment for MetS in obese adults in term of weight loss and decrease of mortality rate. This procedure has been used also in carefully selected obese adolescents and the outcomes seem to be similar to those for adults. However, further studies are needed to better select the patients to surgically treat and define efficacy and safety of bariatric surgery in pediatric MetS.
